# Patients with congenital ichthyosis and *TGM1* mutations overexpress other ARCI genes in the skin: Part of a barrier repair response?

**DOI:** 10.1111/exd.13813

**Published:** 2018-12-21

**Authors:** Hanqian Zhang, Maja Ericsson, Simone Weström, Anders Vahlquist, Marie Virtanen, Hans Törmä

**Affiliations:** ^1^ Department of Medical Sciences, Dermatology and Venereology Uppsala University Uppsala Sweden

**Keywords:** cornified cell envelope, genodermatoses, oligoarray, transcriptome

## Abstract

Autosomal recessive congenital ichthyosis (ARCI) is a group of monogenic skin disorders caused by mutations in any of at least 12 different genes, many of which are involved in the epidermal synthesis of ω‐O‐acylceramides (acylCer). AcylCer are essential precursors of the corneocyte lipid envelope crosslinked by transglutaminase‐1 (TGm‐1), or a yet unidentified enzyme, for normal skin barrier formation. We hypothesized that inactivating *TGM1* mutations will lead to a compensatory overexpression of the transcripts involved in skin barrier repair, including many other ARCI‐causing genes. Using microarray, we examined the global mRNA expression profile in skin biopsies from five ARCI patients with *TGM1* mutations and four healthy controls. There were a total of 599 significantly differentially expressed genes (adjusted *P* < 0.05), out of which 272 showed more than 1.5 log2fold‐change (FC) up‐ or down‐regulation. Functional classification of the latter group of transcripts showed enrichment of mRNA encoding proteins mainly associated with biological pathways involved in keratinocyte differentiation and immune response. Moreover, the expression of seven out of twelve ARCI‐causing genes was significantly increased (FC = 0.98‐2.05). Also, many of the genes involved in keratinocyte differentiation (cornified envelope formation) and immune response (antimicrobial peptides and proinflammatory cytokines) were upregulated. The results from the microarray analysis were also verified for selected genes at the mRNA level by qPCR and at the protein level by semi‐quantitative immunofluorescence. The upregulation of these genes might reflect a compensatory induction of acylCer biosynthesis as a part of a global barrier repair response in the patient′s epidermis.

## 1 INTRODUCTION

Autosomal recessive congenital ichthyosis (ARCI) is a heterogeneous group of rare monogenic skin disorders with a prevalence of 1:100 000.^1^ ARCI is characterized by a defective epidermal barrier reflected in an increased trans‐epidermal water loss (TEWL) and a presumed compensatory hyperkeratosis. At birth, many of these patients present as collodion babies. Subsequently the skin phenotype transforms into either of four major clinical subtypes of ARCI, the most common of which is lamellar ichthyosis.^1^, ^2^, ^3^, ^4^, ^5^ Severe forms of ARCI often cause a reduced quality of life and usually require chronic treatment with systemic retinoids.

ARCI is caused by mutations in any of the following known genes: *TGM1*,* ALOXE3*,* ALOX12B*,* ABCA12*,* CYP4F22*,* CERS3*,* NIPAL4*,* PNPLA1*,* SDR9C7*,* LIPN*,* SLC27A4* and *SULT2B1*.^1^, ^5^, ^6^, ^7^ Additionally, mutations in *ELOVL4*,* GBA*,* GJB2*,* CLDN1*,* SPINK5*,* STS*,* FLG*,* KRT1*,* KRT10* and *KRT2* can cause similar ichthyosis phenotypes.^1^, ^8^, ^9^, ^10^, ^11^, ^12^ Among the first group of genes, the majority are involved in the biosynthesis of ω‐O‐acylceramides (acylCer) and one encodes for an enzyme transglutaminase‐1 (TGm‐1) which cross‐links cornified envelope (CE) proteins and might also be involved in forming the corneocyte lipid envelope (CLE).^1^, ^13^, ^14^, ^15^ The gene products involved in acylCer biosynthesis are described in Supporting Information; the remaining genes referred to above produce various structural proteins and enzymes also essential for skin barrier homoeostasis.

Because TGm‐1 is important in the final step of keratinization and many of the other mentioned gene products are preceding players in the formation of a skin barrier, a genetic deficiency of TGm‐1 could hypothetically elicit compensatory gene regulations either via some pathway‐specific feedback mechanisms or as a part of a more general epidermal repair mechanism.

To address this possibility, which has barely been studied in the past, we examined the global mRNA profile and expression of specific proteins in epidermis of patients with *TGM1* mutations compared with healthy controls.

## 2 MATERIALS AND METHODS

### 2.1 Ethical statement

All studies were approved by the Regional Ethical Review Board (EPN) in Uppsala and conducted according to the Declaration of Helsinki Principles. Informed and written consent was obtained from the patients and healthy controls.

### 2.2 Skin specimens

Skin punch biopsies were taken from the patients and controls (see Supporting Information) for RNA analysis and immunofluorescence staining as previously described.^16^

A detailed description of the patients involved in this study can be found in Table 1 and in a previous report.^4^ In brief, all patients were males, aged 29‐80; four of them were born as collodion babies and later in life required maintenance treatment with oral acitretin, which for ethical reasons could not be stopped to obtain naive skin biopsies for the study. Topical therapy consisted of emollients twice a day which the patients were asked to abandon on gluteal skin for 12 hours before sampling.

**Table 1:** Characteristics of the five ARCI patients with *TGM1* mutations included in the study

Subject	Gender/Age	Mutations^a^	Collodion baby	Scaling^a^	Erythema^a^	Retinoid treatment
P1	M/29	p.[Arg142His];[Gln463His]	No	1	0	No
P2	M/38	p.[Ser358Arg];[Ser358Arg]	Yes	2	0	Yes
P3	M/47	p.[Arg143Cys];[Val379Leu]	Yes	4	1	Yes
P4	M/80	c.[877‐2A>G];[877‐2A>G]	Yes	3	2	Yes
P5	M/46	p.[Ser358Arg];[Ser358Arg]	Yes	2	1	Yes

a The *TGM1* mutations and degree of scaling (0‐4) and erythema (0‐4) have previously been reported.^4^ P2 and P5 are unrelated but carry the same homozygous mutation p.[Ser358Arg] which is common in Sweden and reduces the TGm‐1 activity in vitro to <4%.^41^ The splice site mutation c.[877‐2A>G] in P4 reduces the activity to <7%, and so do the compound heterozygous point mutations in P3.^41^ The point mutation p.[Arg142His] in P1 abolishes the enzymatic activity in vitro, whereas the effect of the p.[Gln463His] mutation has not previously been described, but is presumed to reduce the TGm‐1 activity.

### 2.3 Microarray analysis

GeneChip^®^ ST Arrays (GeneChip^®^ Clariom D Human Array) was used for the microarray analysis of gene expression (see details in Supporting Information). Data were analysed as previously described.^17^

The array data are deposited in Gene Expression Omnibus of NCBI (accession number GSE107462).

### 2.4 Analysis of mRNA expression using qPCR

cDNA was synthesized from total RNA as previously described.^18^, ^19^ Semi‐quantitative PCR was performed using TaqMan Gene Expression Assays (see Table S1 in Supporting Information) in an ABI7500Fast machine. Expression levels were measured in triplicate. The relative mRNA expression was determined by the 2ˆ(−ΔΔCt) method using *RPL19* as a reference gene.

### 2.5 Immunofluorescence (IF)

Patient and normal human skin biopsies were frozen and sectioned at 6 μm in a cryostat. Tissue sections were fixed, blocked, immuno‐stained followed by microphotography and image analysis as described in Supporting Information and Table S2.

### 2.6 Statistical analysis

Statistical methods are described in Supporting Information.

## 3 RESULTS

### 3.1 Transcriptome analysis in patients with *TGM1* mutations versus healthy controls—initial results

**Figure 1:**
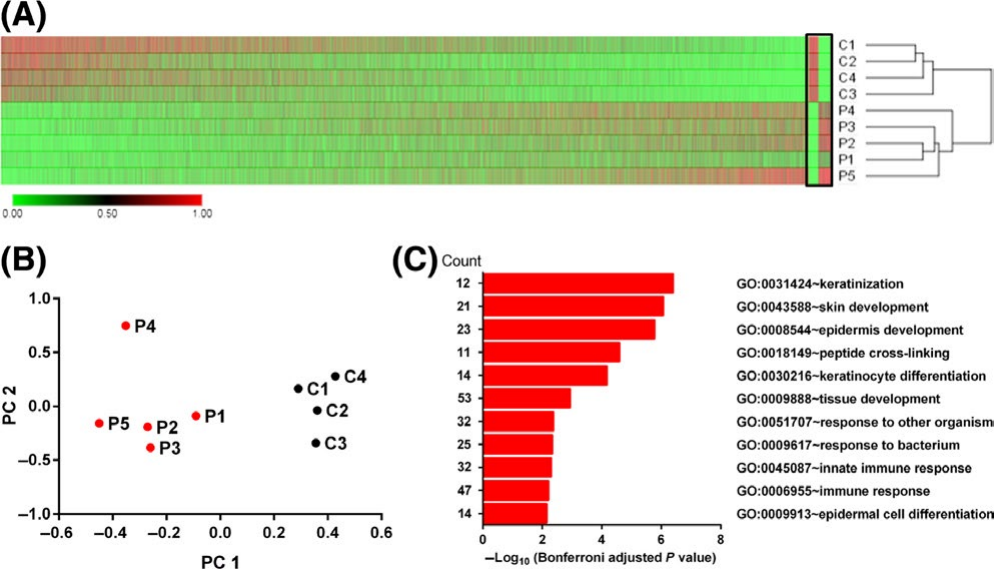
Global gene expression profiles. (A) Heatmap of gene expression profiles (22 559 genes with UniGene ID) of cDNA from skin biopsies of patients with autosomal recessive congenital ichthyosis (ARCI) carrying *TGM1* mutations (P1‐5; n = 5) compared to healthy controls (C1‐4; n = 4) as determined by microarray analysis. The 599 genes that were significantly differentially expressed (adjusted *P* < 0.05) between patients and healthy controls are shown in the black box. Green colour represents relative lower expression and red higher expression. Data for controls and patients were hierarchically clustered together (one minus Pearson correlation distance metric) by MORPHEUS. (B) The data for controls and patients were also separated by principle component analysis (PCA). The principle components (PC) were generated by R program from the global gene expression data. (C) The 272 DEGs (above 1.5 FC and below −1.5 FC) were significantly enriched (Bonferroni adjusted *P* value <0.01) in different gene ontology (GO) terms by functional annotation clustering analysis with DAVID. Each GO term has a certain count number of enriched DEGs

We performed a microarray analysis to investigate the global mRNA expression profile in skin biopsies from five male patients (P1‐5) with ARCI in different severity and various types of inactivating *TGM1* mutations (Table 1) and four healthy controls (aged 39‐68; C1, C2, C4 male and C3 female). Among the transcripts, there were 22 559 genes with UniGene ID, 599 of which were differentially expressed genes (DEGs) (Figure 1A and Table S3). The controls and patients were well separated by two principal components generated from the global expression data (Figure 1B). Out of these DEGs, 205 increased >1.5 log2fold‐change (FC) and 67 decreased <−1.5 FC (which equals a 2.8‐fold change after back‐transformation), making a total of 272 markedly altered transcripts (Table S4) which were functionally enriched in different gene ontology (GO) terms (Figure 1C).

**Figure 2:**
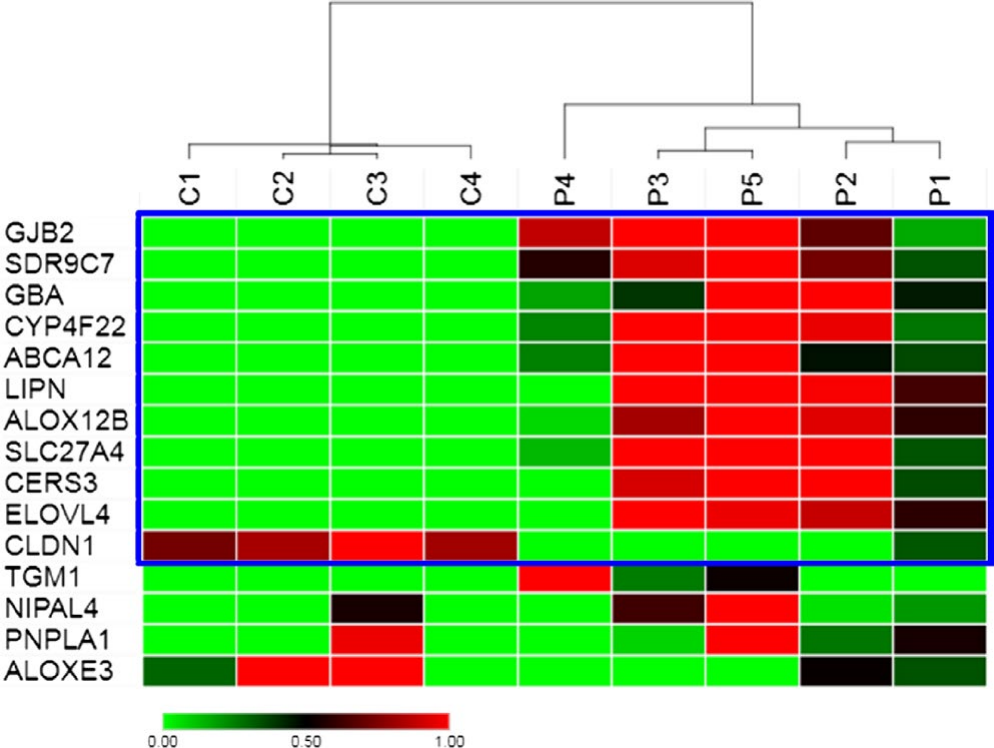
Heatmap of ichthyosis‐related gene expression profile. Heatmap of gene expression profiles of selected ichthyosis‐causing genes in ARCI patients carrying *TGM1* mutations (P1‐5; n = 5) compared to healthy controls (C1‐4; n = 4) as determined by microarray. Genes with significantly changed expression (adjusted *P* < 0.05) between patients and healthy controls are shown in the blue box

A number of DEGs were identified as ichthyosis‐causing and lipid biosynthesis genes (Table S3). For instance, the expression of seven out of twelve ARCI genes was significantly increased, *viz. SDR9C7, CYP4F22, ABCA12*,* LIPN, ALOX12B, SLC27A4* and *CERS3* (0.98‐2.05 FC), as was the expression of three genes causing other types of ichthyosis, *viz. GJB2, GBA* and *ELOVL4* (1.66‐5.69 FC). In contrast, *CLDN1* showed decreased expression (−1.38 FC), and *TGM1, NIPAL4*,* PNPLA1* and *ALOXE3* showed no significant differences compared to controls. Looking at the patients′ individual data (Figure 2), the patterns in P2, P3 and P5 with severe ichthyosis are strikingly similar, whereas P1 showed less pronounced changes consistent with a mild phenotype. The divergent expression pattern in P4 is however more difficult to explain (see Discussion).

With reference to lipid biosynthesis, two genes encoding key enzymes in the early steps of ceramide generation, *SPTLC2* and *SMPD1*, were increased 1.59 and 1.09 FC, respectively (adjusted *P* < 0.054). Moreover, two fatty acid transporters, *CD36* and *FABP5*, were markedly induced (4.12 and 3.84 FC). By contrast, *CERS6*, coding for an enzyme with preference for fatty acyl‐CoA with carbon‐lengths C14‐C18,^20^ was significantly reduced. Also, *SLC27A2,* encoding FATP2, a very‐long‐chain (VLC)‐fatty acyl‐CoA synthase that activates VLC fatty acids (C22‐C24) to their CoA derivatives,^21^ was significantly reduced.

### 3.2 Gene ontology analysis

We used the Database for Annotation, Visualization and Integrated Discovery (DAVID) to examine clusters among the 272 DEGs with adjusted *P* < 0.05 for FC>1.5 or <−1.5 (Table S4). The results show additional clustering of DEGs coding for proteins associated with biological pathways associated with *keratinocyte differentiation* (GO:0030216) and *immune response* (GO:0006955) in Table S5.

Among the DEGs annotated to *keratinocyte differentiation* (also involving the formation of CE), eight small proline‐rich proteins (*SPRRs*) were increased 3.18‐9.31 FC (Table S6). Furthermore, involucrin (*IVL*) and two late cornified envelope proteins (*LCE3E* and *LCE3D*) were induced, as well as *PPARD,* a nuclear ligand‐activated transcription factor known to promote keratinocyte differentiation and lipid metabolism in keratinocytes. In contrast, we did not observe any significant difference in the expression of the keratin genes.

Numerous DEGs were assigned to *immune response* and/or *innate immune response* (GO:0045087) in Table S5 and S7. Among the most strongly induced genes, four coded for calcium binding proteins; *viz. S100A7* (psoriasin), *S100A7A*,* S100A8* and *S100A9* (8.70‐11.29 FC). Also the antimicrobial peptide (AMP) genes *DEFB4A*,* DEFB4B* and *PI3* (encoding SKALP/ELAFIN) were increased 4.16‐9.99 FC. Furthermore, two serine (or cysteine) protease inhibitors, *SERPINB3* (Clade B [Ovalbumin]) and *SERPINB4,* known to be affected in early inflammation and barrier dysfunction,^22^ were among the most strongly induced (8.09 and 7.70 FC, respectively) (Table S3).

Two interleukin genes were significantly changed; *IL36G* (5.13 FC increase) and *IL37* (−3.50 FC decrease), whereas other cytokine genes, for example, *IL1*,* IL17* and *IFNG,* were unaffected. Lastly, three chemokines (*CCL22*,* CCL2* and *CCL20*) with chemotactic activity for several types of inflammatory cells showed marked increases (2.16‐11.64 FC).

### 3.3 Verification by qPCR

**Figure 3:**
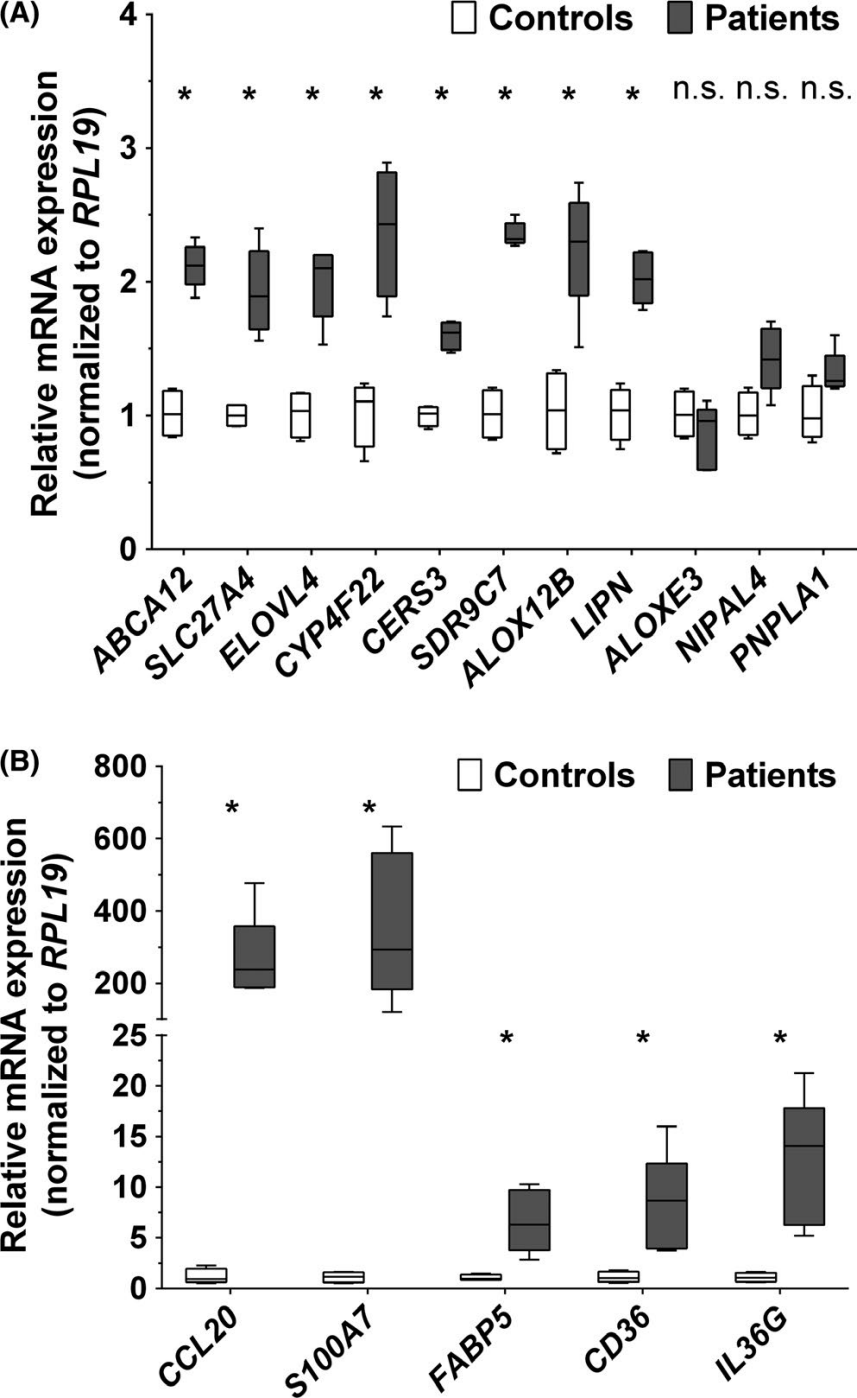
Comparison of selected gene expression by qPCR analysis. Relative mRNA expression of ichthyosis‐causing genes (A) and some other differentially expressed genes (B) in ARCI patients carrying *TGM1* mutations (n = 5) and healthy control subjects (n = 4) analysed by qPCR. Expressions were normalized to the reference gene *RLP19*. Values are presented as box and whiskers with whiskers showing min/max (expression/*RLP19*). *P < 0.05; n.s., not significant

The array data, which indicated changes in the expression of many ichthyosis‐causing and inflammatory‐associated genes, prompted a reinvestigation of the transcripts by qPCR for verification. In total agreement with the array results, mRNA levels of *ABCA12, SLC27A4, ELOVL4, CYP4F22, CERS3, SDR9C7, ALOX12B* and *LIPN* were all increased in patients versus controls, but not so for *ALOXE3*,* NIPAL4* and *PNPLA1* (Figure 3A). In addition, five strongly induced transcripts, but unrelated to any known ARCI aetiology, *viz. CCL20, S100A7, FABP5*,* CD36* and *IL36G,* were markedly increased by qPCR analysis (Figure 3B).

### 3.4 Verification by immunofluorescence staining

**Figure 4:**
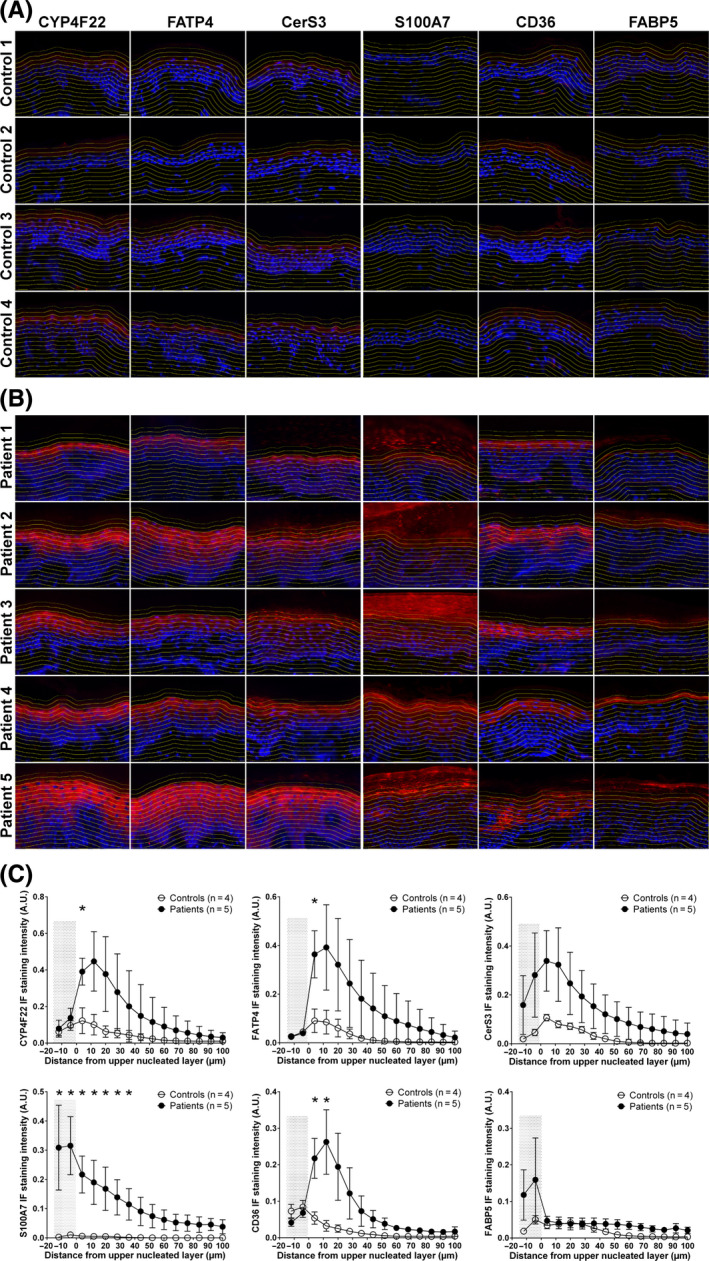
Comparison of protein expression in ARCI patients with *TGM1* mutations (n = 5) with healthy controls (n = 4) by IF staining. The IF staining images of CYP4F22, FATP4, CerS3, S100A7, CD36 and FABP5 in each subject are shown in (A) and (B). The median intensity of IF was measured in each yellow outlined layer representing different stages of epidermal keratinocyte differentiation. The mean intensity for each staining in the patients (B) and controls (A) was plotted in the corresponding graph (C). The outlined layers from top to bottom in the images correspond to the intensity measurements from left −12 to right 100 μm of *X* axis in the graphs, 0 μm indicates the top of upper nucleated layer, and Y axis shows the mean intensity in arbitrary unity (A.U.), and the shadow indicates stratum corneum. Scale bar = 16.1 μm. *P < 0.05

Some genes showing pertinent changes at the transcriptional level were also analysed at the protein level by immunofluorescence (IF) staining of skin sections (Figure 4A and B). By applying image analysis, protein expression—represented by median fluorescence intensity—was also analysed in different epidermal layers using recently developed pipelines for the CellProfiler software^23^ (Figure 4C). It can be seen that the expression of CYP4F22, FATP4 (encoded by *SLC27A4*) and CerS3 in patient skin is markedly increased in the upper spinous and granular layers and also extends to additional epidermal layers compared to control skin (Figure 4A‐C). S100‐A7, which is almost undetectable in control skin, is highly expressed in the patients, especially in the differentiated layers of epidermis (Figure 4). Likewise, the fatty acid transporter CD36 was strikingly increased in the upper spinous layer and granular layer of patient skin (Figure 4), whereas fatty acid binding protein 5 (FABP5) was more variably increased in stratum corneum of some patients compared to controls (Figure 4), thus corroborating our microarray and qPCR results.

## 4 DISCUSSION

This study is one of the first addressing global changes in the cutaneous gene expression in ARCI patients with mutations in *TGM1*. The most pertinent findings are: (a) upregulation of genes involved in the formation of acylCer and CLE, all essential for skin barrier homoeostasis and incriminated in the aetiology of what might be called lipodysgenic ichthyoses; (b) increased expression of various AMPs and cytokine genes involved in antimicrobial response and regulation of immune response; and (c) effects on numerous genes affecting keratinocyte proliferation and differentiation.

### 4.1 Aberrational gene expressions related to lipid biosynthesis and CE/CLE formation

Lipid biosynthesis in epidermis occurs via several pathways essential for a proper barrier function. Our results, showing reduced mRNA expression of *CERS6* and *SLC27A2* involved in the biosynthesis of fatty acids and ceramides with shorter carbon chain length, probably indicate that the production of ultra‐long‐chain fatty acids (acylCer precursors) is prioritized in patients with *TGM1* mutations. In line with this, the expression of *SPTLC2* and *SMPD1* and seven ichthyosis‐causing genes involved in the pathway of acylCer and CLE formation was induced in the range of 2‐4 times. Most of these abnormalities could be verified by qPCR analysis and IF staining of patient and control skin (see Figures 3 and 4).

The TGm‐1 mediated incorporation of proteins into CE is a late, but crucial step in the formation of a skin barrier. Here, we report increased expression in TGm‐1 deficient epidermis of many of the genes encoding CE precursor proteins, such as involucrin (*IVL*), several small proline‐rich proteins (*SPRR1A, SPRR1B, SPRR2A, SPRR2B, SPRR2D, SPRR2E, SPRR2F and SPRR2G*) and late cornified envelope proteins (*LCE3D* and *LCE3E*), which might be considered a positive feedback response.

### 4.2 Effects on genes involved in innate immunity and inflammation

Induced AMP expression has been previously reported in ARCI patients with *TGM1* mutations, as well as in TGm1‐deficient mice.^24^ Our study revealed a number of highly expressed transcripts encoding proteins with antimicrobial activity, *viz*. S100‐A7, S100‐A7A, the S100‐A8/S100‐A9 complex (calprotectin), SKALP/ELAFIN and beta defensin‐4A and 4B. Several of the induced AMP genes may also alter keratinocyte differentiation and proliferation.^25^ Our findings are reminiscent of the situation in lesional psoriatic skin,^25^ except that the expression of Rnase7, LL‐37 and dermcidin was not induced in the ARCI patients with *TGM1* mutations. Typically, neither of these conditions is prone to skin infections despite the barrier abnormalities. However, other subtypes of ichthyoses, that is, harlequin ichthyosis caused by *ABCA12* mutations, Netherton syndrome caused by *SPINK5* mutations and epidermolytic ichthyosis caused by *KRT1* or *KRT10* mutations show increased prevalences of secondary skin infections, which can be attributed both to a more severe barrier failure and to distinctive abnormalities in the lamellar bodies secretory system that collectively decrease the bioavailability of AMPs.^26^

Speculatively, a stimulation of innate immune responses in patient skin might be a consequence of the observed reduction of *IL37*, which is a known suppressor of innate immunity.^27^ Furthermore, an enhanced adaptive immunity, reflected in increased *IL36G* transcription in the array and qPCR analyses, may in turn induce various chemokines and psoriasin (S100‐A7),^28^, ^29^, ^30^ which was also observed in our study.

It is well established that the Th17/IL‐23 pathway is induced in psoriasis^31^ and a previous study of ARCI patients with *TGM1* mutations could link this pathway to ichthyosis severity and inflammation.^32^ Furthermore, an increased IL‐17/IL‐22 activation in peripheral blood in patients with lamellar ichthyosis was shown to correlate with clinical variables in a recent study.^33^ Indeed, we could confirm increased expression of several of the previously reported IL‐17/TNFα‐regulated genes, namely *IL36G/IL1F9, PI3, CCL20, LCN2, DEFB4* and *S100A9*, whereas the expression of the cytokines *IL17A*,* IL17C* and *TNFA* was virtually unchanged.

### 4.3 Effects on other genes related to keratinocyte differentiation

Earlier studies have revealed five genes essential for psoriasis development: *PPARD*,* GATA3*,* TIMP3*,* WNT5A* and *PTTG1*.^34^ In the present study, we also found an altered expression of *PPARD*,* GATA3* and *WNT5A*. As a corollary, we note that a PPARβ/δ agonist has recently been shown to induce *CERS3* and *ELOVL4* in cultured keratinocytes.^35^

### 4.4 Proposed mechanisms for transcriptional changes associated with *TGM1* mutations

The observed upregulation of a multitude of genes in the skin of patients with *TGM1* mutations probably represents a compensatory mechanism for the severe barrier dysfunction elicited by a marked reduction or total lack of TGm‐1 activity. The eliciting factors involved in such a hypothesized compensatory mechanism are still unknown, but increased water flux in stratum corneum,^36^ abnormal signalling by epidermal lipids and a disrupted [Ca^2+^]‐gradient might all be involved. Actually, the latter proposal is supported by the marked induction of *GJB2* observed in the ARCI patients. In the *GJB2‐*deficient mice model for Keratitis‐ichthyosis‐deafness (KID) syndrome, an altered calcium distribution in epidermis results in abnormal lipid processing,^37^ thus implying GJB2 as an essential factor in barrier homoeostasis. Further studies are required to clarify whether the changed expressions of many ARCI genes in patients with *TGM1* mutations are unique for this type of ichthyosis or represents ingredients of a general barrier repair mechanism.

### 4.5 Study limitations

Although this study involved only 5 patients (and 4 controls), the results were in many cases pronounced and principally the same patterns were observed in four of the patients. However, patient P4 (age 80, homozygous for a splice site mutation in *TGM1* and acitretin‐treated for 20 years) showed more equivocal results despite overt lamellar ichthyosis. Incidentally, all patients except P1 were dependent on systemic retinoid therapy that could not be interrupted for a minimum of 1 month required to obtain treatment‐naive skin biopsies. Therefore, we cannot entirely rule out the possibility that some of the observed changes in ARCI‐causing and other genes were affected by retinoids. In an indirect attempt to address this possibility, we exposed human epidermal equivalents (HEE) generated from wild‐type keratinocytes to all‐*trans* retinoic acid (atRA) in vitro (see Supporting Information) and found *reduced* expression of almost all ichthyosis‐causing genes (see Figure S1). This response is similar to previous results in monolayer keratinocytes exposed to atRA^17^ and contradicts retinoid therapy as a cause of the abnormal gene expression changes in the patients. Primary keratinocytes from three of these patients with *TGM1* mutations were isolated for functional studies, but the phenotypic characteristics were lost and problems in generating HEEs using these cells were encountered (see Supporting Information). Furthermore, we previously analysed several retinoid‐regulated genes in healthy volunteers exposed to topical atRA^38^ and lamellar ichthyosis patients treated systemically with the retinoic acid‐metabolism blocking agent, liarozole,^39^ and among all genes found to be upregulated in these reports, only *CRABP2* was upregulated in the ARCI patients of this study. It is worth noting that in a previous study of CRABP protein levels in epidermis, increased expressions were observed in untreated patients with lamellar ichthyosis and psoriasis.^40^ Based on all these evidences, we conclude that acitretin therapy is an unlikely explanation to the transcriptional changes observed in patients P2‐P5.

## 5 CONCLUSION

The present study shows that disruptive mutations in *TGM1* are associated with marked upregulations of several other ichthyosis‐related and lipid biosynthesis genes, as well as modulations of gene expression important for CE formation and immune or inflammatory regulations, together probably constituting a repair mechanism for the epidermal barrier function.

## CONFLICT OF INTEREST

The authors have no conflict of interest to declare.

## AUTHOR CONTRIBUTIONS

HZ, AV, MV and HT contributed to the conception and design of the study. HZ, ME, SW and MV performed the experiments. HZ, ME, AV and HT analysed and interpreted the data. The manuscript was written and reviewed by all authors. All authors read the manuscript and approved the submission.

## References

[1] A. Vahlquist , J. Fischer , H. Törmä , Inherited Nonsyndromic Ichthyoses: An Update on Pathophysiology, Diagnosis and Treatment. Am. J. Clin. Dermatol. 2018, 19, 51. 28815464 10.1007/s40257-017-0313-x PMC5797567

[2] A. Vahlquist , A. Bygum , A. Gånemo , M. Virtanen , M. Hellström‐Pigg , G. Strauss , F. Brandrup , J. Fischer , Genotypic and clinical spectrum of self‐improving collodion ichthyosis: ALOX12B, ALOXE3, and TGM1 mutations in Scandinavian patients. J Invest Dermatol. 2010, 130, 438. 19890349 10.1038/jid.2009.346

[3] E. Bourrat , C. Blanchet‐Bardon , C. Derbois , S. Cure , J. Fischer , Specific TGM1 mutation profiles in bathing suit and self‐improving collodion ichthyoses: phenotypic and genotypic data from 9 patients with dynamic phenotypes of autosomal recessive congenital ichthyosis. Arch. Dermatol. 2012, 148, 1191. 22801880 10.1001/archdermatol.2012.1947

[4] M. Hellström Pigg , A. Bygum , A. Gånemo , M. Virtanen , F. Brandrup , A. D. Zimmer , A. Hotz , A. Vahlquist , J. Fischer , Spectrum of Autosomal Recessive Congenital Ichthyosis in Scandinavia: Clinical Characteristics and Novel and Recurrent Mutations in 132 Patients. Acta Derm. Venereol. 2016, 96, 932. 27025581 10.2340/00015555-2418

[5] T. Takeichi , M. Akiyama , Inherited ichthyosis: Non‐syndromic forms. J. Dermatol. 2016, 43, 242. 26945532 10.1111/1346-8138.13243

[6] L. Heinz , G. J. Kim , S. Marrakchi , J. Christiansen , H. Turki , M. A. Rauschendorf , M. Lathrop , I. Hausser , A. D. Zimmer , J. Fischer , Mutations in SULT2B1 Cause Autosomal‐Recessive Congenital Ichthyosis in Humans. Am. J. Hum. Genet. 2017, 100, 926. 28575648 10.1016/j.ajhg.2017.05.007 PMC5473727

[7] S. Israeli , Z. Khamaysi , D. Fuchs‐Telem , J. Nousbeck , R. Bergman , O. Sarig , E. Sprecher , A mutation in LIPN, encoding epidermal lipase N, causes a late‐onset form of autosomal‐recessive congenital ichthyosis. Am. J. Hum. Genet. 2011, 88, 482. 21439540 10.1016/j.ajhg.2011.02.011 PMC3071911

[8] K. Yoneda , Inherited ichthyosis: Syndromic forms. J. Dermatol. 2016, 43, 252. 26945533 10.1111/1346-8138.13284

[9] M. A. Aldahmesh , J. Y. Mohamed , H. S. Alkuraya , I. C. Verma , R. D. Puri , A. A. Alaiya , W. B. Rizzo , F. S. Alkuraya , Recessive mutations in ELOVL4 cause ichthyosis, intellectual disability, and spastic quadriplegia. Am. J. Hum. Genet. 2011, 89, 745. 22100072 10.1016/j.ajhg.2011.10.011 PMC3234380

[10] E. Basler , M. Grompe , G. Parenti , J. Yates , A. Ballabio , Identification of point mutations in the steroid sulfatase gene of three patients with X‐linked ichthyosis. Am. J. Hum. Genet. 1992, 50, 483. 1539590 PMC1684279

[11] S. Hadj‐Rabia , L. Baala , P. Vabres , D. Hamel‐Teillac , E. Jacquemin , M. Fabre , S. Lyonnet , Y. De Prost , A. Munnich , M. Hadchouel , A. Smahi , Claudin‐1 gene mutations in neonatal sclerosing cholangitis associated with ichthyosis: a tight junction disease. Gastroenterology 2004, 127, 1386. 15521008 10.1053/j.gastro.2004.07.022

[12] N. Tayebi , D. L. Stone , E. Sidransky , Type 2 gaucher disease: an expanding phenotype. Mol. Genet. Metab. 1999, 68, 209. 10527671 10.1006/mgme.1999.2918

[13] M. Akiyama , Corneocyte lipid envelope (CLE), the key structure for skin barrier function and ichthyosis pathogenesis. J. Dermatol. Sci. 2017, 88, 3. 28623042 10.1016/j.jdermsci.2017.06.002

[14] P. M. Elias , M. Schmuth , Y. Uchida , R. H. Rice , M. Behne , D. Crumrine , K. R. Feingold , W. M. Holleran , D. Pharm , Basis for the permeability barrier abnormality in lamellar ichthyosis. Exp. Dermatol. 2002, 11, 248. 12102664 10.1034/j.1600-0625.2001.110308.x

[15] N. Kuramoto , T. Takizawa , T. Takizawa , M. Matsuki , H. Morioka , J. M. Robinson , K. Yamanishi , Development of ichthyosiform skin compensates for defective permeability barrier function in mice lacking transglutaminase 1. J Clin Invest. 2002, 109, 243. 11805136 10.1172/JCI13563 PMC150837

[16] T. Hoppe , M. C. G. Winge , M. Bradley , M. Nordenskjold , A. Vahlquist , B. Berne , H. Törmä , X‐linked recessive ichthyosis: an impaired barrier function evokes limited gene responses before and after moisturizing treatments. Br. J. Dermatol. 2012, 167, 514. 22486194 10.1111/j.1365-2133.2012.10979.x

[17] H. Törmä , A. Bergström , G. Ghiasifarahani , B. Berne , The effect of two endogenous retinoids on the mRNA expression profile in human primary keratinocytes, focusing on genes causing autosomal recessive congenital ichthyosis. Arch. Dermatol. Res. 2014, 306, 739. 24925226 10.1007/s00403-014-1476-4 PMC4168020

[18] I. Buraczewska , B. Berne , M. Lindberg , M. Loden , H. Törmä , Moisturizers change the mRNA expression of enzymes synthesizing skin barrier lipids. Arch. Dermatol. Res. 2009, 301, 587. 19466436 10.1007/s00403-009-0958-2

[19] I. Buraczewska , B. Berne , M. Lindberg , M. Loden , H. Törmä , Long‐term treatment with moisturizers affects the mRNA levels of genes involved in keratinocyte differentiation and desquamation. Arch. Dermatol. Res. 2009, 301, 175. 18850104 10.1007/s00403-008-0906-6

[20] Y. Mizutani , A. Kihara , Y. Igarashi , Mammalian Lass6 and its related family members regulate synthesis of specific ceramides. Biochem J. 2005, 390, 263. 15823095 10.1042/BJ20050291 PMC1184580

[21] E. M. Melton , R. L. Cerny , C. C. DiRusso , P. N. Black , Overexpression of human fatty acid transport protein 2/very long chain acyl‐CoA synthetase 1 (FATP2/Acsvl1) reveals distinct patterns of trafficking of exogenous fatty acids. Biochem. Biophys. Res. Commun. 2013, 440, 743. 24113382 10.1016/j.bbrc.2013.09.137 PMC4665974

[22] U. Sivaprasad , K. G. Kinker , M. B. Ericksen , M. Lindsey , A. M. Gibson , S. A. Bass , N. S. Hershey , J. Deng , M. Medvedovic , G. K. Khurana Hershey , SERPINB3/B4 contributes to early inflammation and barrier dysfunction in an experimental murine model of atopic dermatitis. J Invest Dermatol. 2015, 135, 160. 25111616 10.1038/jid.2014.353 PMC4268075

[23] H. Zhang , M. Ericsson , M. Virtanen , S. Weström , C. Wählby , A. Vahlquist , H. Törmä , Quantitative image analysis of protein expression and colocalisation in skin sections. Exp. Dermatol. 2018, 27, 196. 29094393 10.1111/exd.13457

[24] T. Haneda , Y. Imai , R. Uchiyama , O. Jitsukawa , K. Yamanishi , Activation of Molecular Signatures for Antimicrobial and Innate Defense Responses in Skin with Transglutaminase 1 Deficiency. PLoS ONE 2016, 11, e0159673. 27442430 10.1371/journal.pone.0159673 PMC4956052

[25] F. Niyonsaba , C. Kiatsurayanon , P. Chieosilapatham , H. Ogawa , Friends or Foes? Host defense (antimicrobial) peptides and proteins in human skin diseases. Exp. Dermatol. 2017, 26, 989. 28191680 10.1111/exd.13314

[26] A. Chan , E. Godoy‐Gijon , A. Nuno‐Gonzalez , D. Crumrine , M. Hupe , E. H. Choi , R. Gruber , M. L. Williams , K. Choate , P. H. Fleckman , P. M. Elias , Cellular basis of secondary infections and impaired desquamation in certain inherited ichthyoses. JAMA Dermatol. 2015, 151, 285. 25565224 10.1001/jamadermatol.2014.3369 PMC4498571

[27] M. F. Nold , C. A. Nold‐Petry , J. A. Zepp , B. E. Palmer , P. Bufler , C. A. Dinarello , IL‐37 is a fundamental inhibitor of innate immunity. Nat. Immunol. 2010, 11, 1014. 20935647 10.1038/ni.1944 PMC3537119

[28] N. Li , K. Yamasaki , R. Saito , S. Fukushi‐Takahashi , R. Shimada‐Omori , M. Asano , S. Aiba , Alarmin Function of Cathelicidin Antimicrobial Peptide LL37 through IL‐36 gamma Induction in Human Epidermal Keratinocytes. J Immunol. 2014, 193, 5140. 25305315 10.4049/jimmunol.1302574

[29] A. M. Foster , J. Baliwag , C. S. Chen , A. M. Guzman , S. W. Stoll , J. E. Gudjonsson , N. L. Ward , A. Johnston , IL‐36 promotes myeloid cell infiltration, activation, and inflammatory activity in skin. J Immunol. 2014, 192, 6053. 24829417 10.4049/jimmunol.1301481 PMC4048788

[30] A. M. D'Erme , D. Wilsmann‐Theis , J. Wagenpfeil , M. Holzel , S. Ferring‐Schmitt , S. Sternberg , M. Wittmann , B. Peters , A. Bosio , T. Bieber , J. Wenzel , IL‐36 gamma (IL‐1F9) Is a Biomarker for Psoriasis Skin Lesions. J Invest Dermatol. 2015, 135, 1025. 25525775 10.1038/jid.2014.532

[31] E. Guttman‐Yassky , J. G. Krueger , M. G. Lebwohl , Systemic immune mechanisms in atopic dermatitis and psoriasis with implications for treatment. Exp. Dermatol. 2018, 27, 409. 28266782 10.1111/exd.13336

[32] A. S. Paller , Y. Renert‐Yuval , M. Suprun , H. Esaki , M. Oliva , T. N. Huynh , B. Ungar , N. Kunjravia , R. Friedland , X. Peng , X. Zheng , Y. D. Estrada , J. G. Krueger , K. A. Choate , M. Suarez‐Farinas , E. Guttman‐Yassky , An IL‐17‐dominant immune profile is shared across the major orphan forms of ichthyosis. J Allergy Clin Immunol. 2017, 139, 152. 27554821 10.1016/j.jaci.2016.07.019 PMC8033419

[33] T. Czarnowicki , H. He , A. Leonard , K. Malik , S. Magidi , S. Rangel , K. Patel , K. Ramsey , M. Murphrey , T. Song , Y. Estrada , H. C. Wen , J. G. Krueger , E. Guttman‐Yassky , A. S. Paller , The Major Orphan Forms of Ichthyosis Are Characterized by Systemic T‐Cell Activation and Th‐17/Tc‐17/Th‐22/Tc‐22 Polarization in Blood. J Invest Dermatol. 2018, 138, 2157. 29660300 10.1016/j.jid.2018.03.1523

[34] J. Dou , L. Zhang , X. Xie , L. Ye , C. Yang , L. Wen , C. Shen , C. Zhu , S. Zhao , Z. Zhu , B. Liang , Z. Wang , H. Li , X. Fan , S. Liu , X. Yin , X. Zheng , L. Sun , S. Yang , Y. Cui , F. Zhou , X. Zhang , Integrative analyses reveal biological pathways and key genes in psoriasis. Br. J. Dermatol. 2017, 177, 1349. 28542811 10.1111/bjd.15682

[35] Y. Mizutani , H. Sun , Y. Ohno , T. Sassa , T. Wakashima , M. Obara , K. Yuyama , A. Kihara , Y. Igarashi , Cooperative Synthesis of Ultra Long‐Chain Fatty Acid and Ceramide during Keratinocyte Differentiation. PLoS ONE 2013, 8, e67317. 23826266 10.1371/journal.pone.0067317 PMC3694974

[36] G. Grubauer , P. M. Elias , K. R. Feingold , Transepidermal water loss: the signal for recovery of barrier structure and function. J. Lipid Res. 1989, 30, 323. 2723540

[37] F. Bosen , A. Celli , D. Crumrine , K. vom Dorp , P. Ebel , H. Jastrow , P. Dormann , E. Winterhager , T. Mauro , K. Willecke , Altered epidermal lipid processing and calcium distribution in the KID syndrome mouse model Cx26S17F. FEBS Lett. 2015, 589, 1904. 26070424 10.1016/j.febslet.2015.05.047 PMC4741282

[38] M. Virtanen , H. Törmä , A. Vahlquist , Keratin 4 upregulation by retinoic acid in vivo: a sensitive marker for retinoid bioactivity in human epidermis. J Invest Dermatol. 2000, 114, 487. 10692107 10.1046/j.1523-1747.2000.00901.x

[39] E. Pavez Loriè , A. Gånemo , M. Borgers , L. Wouters , S. Blockhuys , L. van de Plassche , H. Törmä , A. Vahlquist , Expression of retinoid‐regulated genes in lamellar ichthyosis vs. healthy control epidermis: changes after oral treatment with liarozole. Acta Derm. Venereol. 2009, 89, 12. 19197536 10.2340/00015555-0573

[40] G. Siegenthaler , J. H. Saurat , D. Salomon , Y. Merot , Skin cellular retinoid‐binding proteins and retinoid‐responsive dermatoses. Dermatologica. 1986, 173, 163. 2429879 10.1159/000249244

[41] M. Huber , V. C. Yee , N. Burri , E. Vikerfors , A. P. Lavrijsen , A. S. Paller , D. Hohl , Consequences of seven novel mutations on the expression and structure of keratinocyte transglutaminase. J. Biol. Chem. 1997, 272, 21018. 9261103 10.1074/jbc.272.34.21018

