# Supplementary material for: Patients with congenital ichthyosis and TGM1 mutations overexpress other ARCI genes in the skin: Part of a barrier repair response?
Source: Exp Dermatol. 2018 Dec 21;28(10):1164–71. doi: 10.1111/exd.13813 (PMC7379499; doi:10.1111/exd.13813)
Supplement: Supplementary file 2 [file EXD-28-1164-s002.docx]

**Supporting Information**

**Supplementary Introduction**

**Biosynthesis of ω-O-acylceramides (acylCer) and formation of corneocyte lipid envelope (CLE)**

ω-O-acylceramides (acylCer) consist of an ultra-long chain (C28-C36) fatty acid (ULCFA), sphingosine and a ω-esterified linoleic acid ^[1]^. The following enzymes/transport proteins participate in the acylCer biosynthesis: ELOVL4 (ELOVL fatty acid elongase 4) required for ULCFA synthesis ^[2,3]^, CYP4F22 (cytochrome P450 family 4 subfamily F member 22) responsible for the hydroxylation of the ω-carbon in the ULCFA ^[1]^, FATP4 (Fatty acid transporter protein 4), encoded by the *SLC27A4* gene, a putative acyl-CoA synthetase activating ULCFA ^[4-6]^, which then serves as a substrate for CerS3 (ceramide synthase 3) catalyzing an amide bond between sphingosine and ULCFA, producing ω-hydroxyl ceramide (ω-OH-Cer) ^[1,6,7]^. Furthermore, PNPLA1 (Patatin-like phospholipase domain containing 1) is required for esterification of ω-OH-Cer with linoleic acid at the ω-carbon to generate acylCer ^[8-10]^.

After glycosylation, acylCer is transferred to the lamellar bodies for storage with assistance of a lipid transport protein, ABCA12 (ATP-binding cassette (ABC) sub-family A member 12), and is eventually released to the extracellular space via exocytosis ^[6,11,12]^. Here, the linoleate moiety of acylCer is oxygenated by 12(R)-lipoxygenase (12R-LOX) and lipoxygenase-3 (eLOX3) encoded by the *ALOX12B* and *ALOXE3* genes, respectively ^[6,13-17]^. Transglutaminase-1 (TGm-1), a cross-linking enzyme encoded by *TGM1,* probably serves the dual function of first forming cornified cell envelope (CE) from precursors such as small proline-rich proteins (SPRRs), late cornified envelope proteins (LCEs), loricrin and involucrin ^[18]^, and hence covalently binding acylCer to the surface of CE, constituting the corneocyte lipid envelope (CLE) ^[6,15,17-19]^. In a last step, glycosylated acylCer is deglycosylated by β-glucocerebrosidase, encoded by *GBA*, a deficiency of which causes Gaucher disease sometimes associated with ichthyosis ^[6,20,21]^.

**Supplementary Materials and Methods**

**Skin samples**

After informed and written consent and local anesthesia, 3 mm punch biopsies were obtained from lesioned skin on the trunk of the patients and controls. In brief, all patients were males, aged 29-84; four of them were born as collodion babies and later in life required maintenance treatment with oral acitretin, an aromatic retinoid, which for ethical reasons could not be stopped to obtain naive skin biopsies for the study. Topical therapy consisted of emollients twice a day which the patients were asked to abandon on gluteal skin for 12 hours before sampling. Our controls are 3 males (C1, C2, C4) and 1 female (C3), aged 39-68.

**Microarray expression analysis**

GeneChip® ST Arrays (GeneChip® Clariom D Human Array) was used for the microarray analysis of gene expression. RNA is isolated by using TRI Reagent (Ambion, Thermo Fisher Scientific) from homogenized skin biopsies and RNA quality was evaluated by Agilent 2100 Bioanalyzer system (Agilent Technologies Inc, Palo Alto, CA). 250 ng of total RNA from each sample was used to generate amplified and biotinylated sense-strand cDNA from the entire expressed genome according to the GeneChip® WT PLUS Reagent Kit User Manual (P/N 703174, Thermo Fisher Scientific Inc., Waltham, MA). GeneChip® ST Arrays (GeneChip® Clariom D Human Array) were hybridized for 16 hours in a 45°C incubator, rotated at 60 rpm. According to the GeneChip® Expression Wash, Stain and Scan Manual (P/N 702731, Thermo Fisher Scientific Inc., Waltham, MA) the arrays were then washed and stained using the Fluidics Station 450 and finally scanned using the GeneChip® Scanner 3000 7G.

Raw data was normalized in the free software Expression Console provided by Affymetrix (http://www.affymetrix.com) by using the robust multi-array average (RMA) method ^[22,23]^. The RMA algorithm fits a robust linear model at the probe level to minimize the effect of probe-specific affinity differences. Analysis of the gene expression data were carried out in the freely available statistical computing language R (http://www.rproject.org) using packages available from the Bioconductor project (www.bioconductor.org). In order to search for genes that were differentially expressed in different groups, an empirical Bayes’ moderated t test was applied ^[24]^, using the ‘limma’ package ^[25]^. To address the problem with multiple testing, the p values were adjusted using the method of Benjamini and Hochberg ^[26]^.

Heat maps of gene expression profile were generated using the online tool Morpheus (https://software.broadinstitute.org/morpheus). Principle components (PC) were generated by using R program from global gene expression data. The most two distinguishable PCs were plotted to separate the samples. Gene expressions which differed more than 1.5 log2fold-change (FC) compared to controls (adjusted P<0.05) was used in functional annotation clustering analysis with the Database for Annotation, Visualization and Integrated Discovery (DAVID) ^[22,27]^. Gene ontology clusters with Bonferroni adjusted P value<0.01 were retained for evaluation. The array data are deposited in the Gene Expression Omnibus of the National Center for Biotechnology Information (NCBI) and are accessible through its series accession number GSE107462.

**Analysis of mRNA expression using qPCR**

First-strand cDNA was synthesized from 150 ng total RNA by combining oligo(d)T_15_, random hexamers, buffer and MMLV reverse transcriptase (Invitrogen) as previously described ^[28,29]^. Semi-quantitative PCR was performed using cDNA (equivalent to 2.5 ng total RNA) as template and TaqMan Gene Expression Assays (Invitrogen; see Table S1 for detailed information) in an ABI7500Fast PCR machine (Applied Biosystems). Expression levels were measured in triplicate. The relative mRNA expression was determined by the 2ˆ(-ΔΔCt) method using *RPL19* as a reference gene.

**Table S1.** TaqMan qPCR detection primers and probes used.

| **Gene** | **GX set #** | **Product Size (bp)** |
| --- | --- | --- |
| **Reference genes** |  |  |
| *RPL19* | Hs01577060_gH | 142 |
| **Genes of interest** |  |  |
| *S100A7* | Hs01923188_u1 | 178 |
| *CCL20* | Hs00355476_m1 | 70 |
| *CD36* | Hs00354519_m1 | 83 |
| *FABP5* | Hs02339439_g1 | 91 |
| *IL36G* | Hs00219742_m1 | 64 |
| *SDR9C7* | Hs00541011_m1 | 64 |
| *CYP4F22* | Hs00403446_m1 | 81 |
| *ELOVL4* | Hs00224122_m1 | 80 |
| *CERS3* | Hs00698859_m1 | 99 |
| *ABCA12* | Hs00292421_m1 | 77 |
| *SLC27A4* | Hs00192700_m1 | 75 |
| *ALOX12B* | Hs00153961_m1 | 73 |
| *LIPN* | Hs01037830_m1 | 87 |
| *NIPAL4* | Hs00398027_m1 | 76 |
| *ALOXE3* | Hs01107034_m1 | 80 |
| *PNPLA1* | Hs00543592_m1 | 75 |

Source: ThermoFisher Scientific.

**Immunofluorescence (IF)**

Tissue sections (6µm) were fixed in ice-cold acetone for 10 minutes, air-dried and washed followed by addition of Background Sniper (Biocare Medical) for 15 minutes at room temperature (RT). The tissue samples were then incubated at 4°C overnight with primary antibodies (see Table S2 for detailed information). After rinsing, the specimens were incubated with biotinylated horse anti-mouse or goat anti-rabbit secondary antibody (Vector Labs, Burlingame, USA) diluted 1:200 for 1 hour. Following washing, the sections were incubated in the dark with TexasRed-labelled Avidin D (Vector Labs) at a 1:500 dilution at room temperature for 30 minutes. The washed slides were mounted with Vectashield containing 4’,6-diamidino-2-phenylindole (DAPI) (Vector Labs) and analyzed with an AxioImager Z1 (Carl Zeiss, Stockholm, Sweden). Phosphate-buffered saline (PBS, Sigma) was used for washing, and 2% bovine serum albumin (BSA) in PBS for dissolving the antibodies and fluorescent reagent.

For each section, at least six microphotographs were obtained with a Zeiss AxioImager Z1 microscope (Carl Zeiss, Stockholm, Sweden) at 40x magnification using the ZEN2012 software. The median fluorescence intensities in various layers of the sections were recorded using the CellProfiler software ^[30]^ and recently reported pipelines (http://cellprofiler.org/examples/published_pipelines) ^[31]^. The mean of the median intensities for each layer and subject was determined. The average +/- SD for patients and controls was then calculated.

**Table S2.** Primary antibodies used.

| **Antibody** | **Species** | **Dilution** | **Cat no.** | **Source** |
| --- | --- | --- | --- | --- |
| Anti-S100A7 | Mouse/monoclonal | 1/200 | ab13680 | Abcam plc, Cambridge, UK |
| Anti-CD36 | Mouse/monoclonal | 1/100 | ab17044 | Abcam plc, Cambridge, UK |
| Anti-FABP5 | Rabbit/polyclonal | 1/200 | ab84028 | Abcam plc, Cambridge, UK |
| Anti-FATP4 | Mouse/monoclonal | 1/200 | H00010999-M01 | Abnova, Taipei, Taiwan |
| Anti-CYP4F22 | Mouse/polyclonal | 1/50 | H00126410-B01P | Abnova, Taipei, Taiwan |
| Anti-CERS3 | Rabbit/polyclonal | 1/100 | HPA006092 | Sigma, Stockholm, Sweden |

**Human epidermal equivalents (HEE)**

Primary human epidermal keratinocytes of neonatal origin (Life Technologies) were cultured in EpiLife medium with supplements (Life Technologies). After trypsinisation, 300,000 cells were seeded per 12mm insert (0.4µm pore size, Merck Millipore) in EpiLife medium and incubated as submerged culture for 48h. Then the medium was changed to CnT-Prime 3D Barrier medium (CellnTec) supplemented with 20% DMEM (Sigma) (called: CnT/DMEM) and incubated for another 24h in submerged culture. In order to lift the inserts to the air-liquid interface, the medium inside was removed and the inserts were transferred to 6cm dishes with 3.2ml CnT/DMEM (3-4 inserts per dish). After 12 days at the air-liquid interface, the HEE were treated with 1µM *all-trans* retinoic acid (atRA) (Sigma) or vehicle DMSO for 24h, 48h or 72h. During these three days, medium or treatment was changed every day. Then, the HEE were harvested in TRI Reagent (Ambion, Thermo Fisher Scientific), mRNA was isolated and subsequently used for qPCR analysis.

Primary keratinocytes from three of these patients with *TGM1*-mutations were isolated for functional studies, but the phenotypic characteristics were lost and the problem of premature keratinocyte differentiation in generating HEEs using these cells was encountered. To circumvent this problem, larger biopsies are needed but this is not possible due to ethical reasons.

**Statistical analysis**

The microarray data were analyzed using Bayes´moderated t test as described above. The results of qPCR and IF analyses were analyzed using the Holm-Sidak method for multiple t-test with α=5% (Prism 6.05 software, GraphPad Software Inc., La Jolla, CA). Since the sample size was too small to determine the normality of the data, multiple Mann-Whitney rank tests were also performed, and no significant difference was found between patients and controls after Bonferroni adjustment of P values.

**Supplementary Results**





**Figure S1.** qPCR analysis of gene expression of ichthyosis-causing genes in HEEs of normal human epidermal keratinocytes exposed to atRA (1 µM) for 3 days. The expression of almost all significantly upregulated ichthyosis-causing genes in ARCI patients with *TGM1* mutations were reduced in HEEs at 3-day exposure to atRA. Relative mRNA expression was normalized to *RLP19*. Values are presented as mean with SD (expression/*RLP19*). *P <0.05.

**References**

[1] Y. Ohno, S. Nakamichi, A. Ohkuni, et al. Essential role of the cytochrome P450 CYP4F22 in the production of acylceramide, the key lipid for skin permeability barrier formation. *Proc Natl Acad Sci U S A.* **2015**;112(25):7707-7712.

[2] M. P. Agbaga, R. S. Brush, M. N. Mandal, K. Henry, M. H. Elliott, R. E. Anderson. Role of Stargardt-3 macular dystrophy protein (ELOVL4) in the biosynthesis of very long chain fatty acids. *Proc Natl Acad Sci U S A.* **2008**;105(35):12843-12848.

[3] A. Kihara. Synthesis and degradation pathways, functions, and pathology of ceramides and epidermal acylceramides. *Prog Lipid Res.* **2016**;63:50-69.

[4] Z. Z. Jia, C. L. Moulson, Z. T. Pei, J. H. Miner, P. A. Watkins. Fatty acid transport protein 4 is the principal very long chain fatty Acyl-CoA synthetase in skin fibroblasts. *J Biol Chem.* **2007**;282(28):20573-20583.

[5] H. Li, A. Vahlquist, H. Törmä. Interactions between FATP4 and ichthyin in epidermal lipid processing may provide clues to the pathogenesis of autosomal recessive congenital ichthyosis. *J Dermatol Sci.* **2013**;69(3):195-201.

[6] M. Akiyama. Corneocyte lipid envelope (CLE), the key structure for skin barrier function and ichthyosis pathogenesis. *J Dermatol Sci.* **2017**;88(1):3-9.

[7] R. Jennemann, M. Rabionet, K. Gorgas, et al. Loss of ceramide synthase 3 causes lethal skin barrier disruption. *Hum Mol Genet.* **2012**;21(3):586-608.

[8] S. Grond, T. O. Eichmann, S. Dubrac, et al. PNPLA1 Deficiency in Mice and Humans Leads to a Defect in the Synthesis of Omega-O-Acylceramides. *J Invest Dermatol.* **2017**;137(2):394-402.

[9] T. Hirabayashi, T. Anjo, A. Kaneko, et al. PNPLA1 has a crucial role in skin barrier function by directing acylceramide biosynthesis. *Nat Commun.* **2017**;8:14609.

[10] Y. Ohno, N. Kamiyama, S. Nakamichi, A. Kihara. PNPLA1 is a transacylase essential for the generation of the skin barrier lipid omega-O-acylceramide. *Nat Commun.* **2017**;8:14610.

[11] M. Akiyama, Y. Sugiyama-Nakagiri, K. Sakai, et al. Mutations in lipid transporter ABCA12 in harlequin ichthyosis and functional recovery by corrective gene transfer. *J Clin Invest.* **2005**;115(7):1777-1784.

[12] M. Akiyama. The roles of ABCA12 in epidermal lipid barrier formation and keratinocyte differentiation. *Biochim Biophys Acta Mol Cell Biol Lipids.* **2014**;1841(3):435-440.

[13] N. Epp, G. Furstenberger, K. Muller, et al. 12R-lipoxygenase deficiency disrupts epidermal barrier function. *J Cell Biol.* **2007**;177(1):173-182.

[14] J. L. Moran, H. Qiu, A. Turbe-Doan, et al. A mouse mutation in the 12R-lipoxygenase, Alox12b, disrupts formation of the epidermal permeability barrier. *J Invest Dermatol.* **2007**;127(8):1893-1897.

[15] Y. Zheng, H. Yin, W. E. Boeglin, et al. Lipoxygenases mediate the effect of essential fatty acid in skin barrier formation: a proposed role in releasing omega-hydroxyceramide for construction of the corneocyte lipid envelope. *J Biol Chem.* **2011**;286(27):24046-24056.

[16] P. Krieg, S. Rosenberger, S. de Juanes, et al. Aloxe3 Knockout Mice Reveal a Function of Epidermal Lipoxygenase-3 as Hepoxilin Synthase and Its Pivotal Role in Barrier Formation. *J Invest Dermatol.* **2013**;133(1):172-180.

[17] A. Dick, I. Tantcheva-Poor, V. Oji, et al. Diminished protein-bound omega-hydroxylated ceramides in the skin of patients with ichthyosis with 12R-lipoxygenase (LOX) or eLOX-3 deficiency. *Br J Dermatol.* **2017**;177(4):e119-e121.

[18] Z. Nemes, L. N. Marekov, L. Fesus, P. M. Steinert. A novel function for transglutaminase 1: attachment of long-chain omega-hydroxyceramides to involucrin by ester bond formation. *Proc Natl Acad Sci U S A.* **1999**;96(15):8402-8407.

[19] A. Munoz-Garcia, C. P. Thomas, D. S. Keeney, Y. Zheng, A. R. Brash. The importance of the lipoxygenase-hepoxilin pathway in the mammalian epidermal barrier. *Biochim Biophys Acta.* **2014**;1841(3):401-408.

[20] W. M. Holleran, E. I. Ginns, G. K. Menon, et al. Consequences of beta-glucocerebrosidase deficiency in epidermis. Ultrastructure and permeability barrier alterations in Gaucher disease. *J Clin Invest.* **1994**;93(4):1756-1764.

[21] N. Tayebi, D. L. Stone, E. Sidransky. Type 2 gaucher disease: an expanding phenotype. *Mol Genet Metab.* **1999**;68(2):209-219.

[22] R. A. Irizarry, B. Hobbs, F. Collin, et al. Exploration, normalization, and summaries of high density oligonucleotide array probe level data. *Biostatistics.* **2003**;4(2):249-264.

[23] C. Li, W. H. Wong. Model-based analysis of oligonucleotide arrays: expression index computation and outlier detection. *Proc Natl Acad Sci U S A.* **2001**;98(1):31-36.

[24] G. K. Smyth. Linear models and empirical bayes methods for assessing differential expression in microarray experiments. *Stat Appl Genet Mol Biol.* **2004**;3:Article3.

[25] G. K. Smyth. Limma: linear models for microarray data. In: R. Gentleman, V. Carey, S. Dudoit, R. Irizarry, W. Huber, eds. *Bioinformatics and Computational Biology Solutions using R and Bioconductor.* New York: Springer; **2005**:397-420.

[26] Y. Benjamini, Y. Hochberg. Controlling the False Discovery Rate - a Practical and Powerful Approach to Multiple Testing. *J Roy Stat Soc B Met.* **1995**;57(1):289-300.

[27] D. W. Huang, B. T. Sherman, R. A. Lempicki. Systematic and integrative analysis of large gene lists using DAVID bioinformatics resources. *Nat Protoc.* **2009**;4(1):44-57.

[28] I. Buraczewska, B. Berne, M. Lindberg, M. Loden, H. Törmä. Moisturizers change the mRNA expression of enzymes synthesizing skin barrier lipids. *Arch Dermatol Res.* **2009**;301(8):587-594.

[29] I. Buraczewska, B. Berne, M. Lindberg, M. Loden, H. Törmä. Long-term treatment with moisturizers affects the mRNA levels of genes involved in keratinocyte differentiation and desquamation. *Arch Dermatol Res.* **2009**;301(2):175-181.

[30] L. Kamentsky, T. R. Jones, A. Fraser, et al. Improved structure, function and compatibility for CellProfiler: modular high-throughput image analysis software. *Bioinformatics.* **2011**;27(8):1179-1180.

[31] H. Zhang, M. Ericsson, M. Virtanen, et al. Quantitative image analysis of protein expression and colocalisation in skin sections. *Exp Dermatol.* **2018**;27(2):196-199.
